# Metabolomic studies as a tool for determining the post-mortem interval (PMI) in stillborn calves

**DOI:** 10.1186/s12917-019-1935-4

**Published:** 2019-06-07

**Authors:** Paulina Jawor, Adam Ząbek, Wojciech Wojtowicz, Dawid Król, Tadeusz Stefaniak, Piotr Młynarz

**Affiliations:** 1 Department of Immunology, Pathophysiology and Veterinary Preventive Medicine, Wroclaw University of Environmental and Life Sciences, Wroclaw, Poland; 2 Department of Bioorganic Chemistry, Wroclaw University of Technology, Wroclaw, Poland

**Keywords:** Stillborn calf, Metabolomics, Molecular diagnostics, Veterinary science, Nuclear magnetic resonance

## Abstract

**Background:**

Perinatal mortality may vary between herds, but the cost of deaths are always higher than value of the calf. When diagnosing the cause of a calf’s death it is important to determine when it occurred, before or after calving. Metabolomics is widely used to identify many human diseases, but quite rarely applied in veterinary science. The aim of this study was to compare the metabolic profiles of calves with different times of death and those of calves born alive. Into the study, twenty one healthy controls (singleton, normal assisted calving, born alive) and 75 stillborn (SB) calves (with a gestation length of ≥260 days, SB, or dead within 6 h of birth) were enrolled. Plasma and urine from SB and control calves were investigated by proton nuclear magnetic resonance based metabolomic methods. SB calves were divided into four PMI groups. One PMI group included calves that died after calving and the other groups - three comprised in utero deaths, based on pathophysiological changes (lung inflation, autolysis in internal organs, hemoglobin imbibition in the pleura and aortic arch). Partial Least Squares - Discriminant Analysis models based on plasma metabolites were calculated, reflecting assumed data clustering.

**Results:**

Twenty six metabolites in plasma and 29 in urine changed significantly with PMI according to one way analysis of variance. Half the metabolites in plasma and the majority in urine increased with PMI. Six metabolites increased simultaneously in plasma and urine: acetate, sn-glycero-3-phosphocholine (GPC), leucine, valine, creatine, and alanine.

**Conclusions:**

Post-mortem changes in calves were associated with molecular variations in blood plasma and urine, showing the greatest differences for the group in which the post-mortem pathological changes were the most advanced. The results of the study show that evaluation of calf plasma or urine may be used as a diagnostic method for the determination of the PMI. Moreover, the metabolites, which unambiguously increased or decreased, can be used as potential biomarkers of PMI.

**Electronic supplementary material:**

The online version of this article (10.1186/s12917-019-1935-4) contains supplementary material, which is available to authorized users.

## Background

The average gestation length in Holstein-Friesian cattle is 279.5 (±5) days [1]. If the calf is born dead or dies within 24 h after birth, after at least 260 days of gestation, then it is classified as stillborn (SB) [2]. Perinatal mortality rates may vary; some herds have 0% SB cases, while others have 30.6% [3]. Losses from stillbirth are far greater than just the value of the calf. Cows that have a stillbirth have a significantly increased risk of culling/death throughout the lactation period and exhibit an increase of 88 days in median days open compared with cows that had live calves [4]. The cause of death may be classified as either non-infectious (most common in cases of difficult calving) or infectious (less common). Identifying the cause of death is especially difficult in cases where calves die in utero [2].

Necropsy examination is critical for determining the time and cause of death, which could be a crucial factor for herd profitability. The degree of carcass autolysis has been used so far to estimate the duration of retention in utero following foetal death, but these estimates are based on sterile foetal autolysis in calves, lambs, and piglets [5].

Despite many efforts to anticipate foetal calf health, until now no unequivocal protein molecular marker has been found in either the cow or the calf. Therefore, another approach may be applied by using the metabolomics methods. Based on this methodology, two types of molecular diagnostics can be performed. One uses the search for and identification of specific low molecular weight biomarkers. The second, metabolic profiling with the application of the ^1^H NMR method, uses the pool of all metabolites as input to create a predictive diagnostics model. With the use of chemometric and statistical methods, the pattern of molecular-level changes between the reference and investigated groups is recognized. This approach is used widely to identify many human diseases [6], but quite rarely used in veterinary science. We hypothesized that the metabolic profile of a calf at calving would vary depending on the time of death or if it was a live birth, therefore metabolomics can be used as a tool for evaluating the post-mortem interval (PMI).

## Results

### Post-mortem calf stratification, chemometric, and statistical analysis

**Fig. 1:**
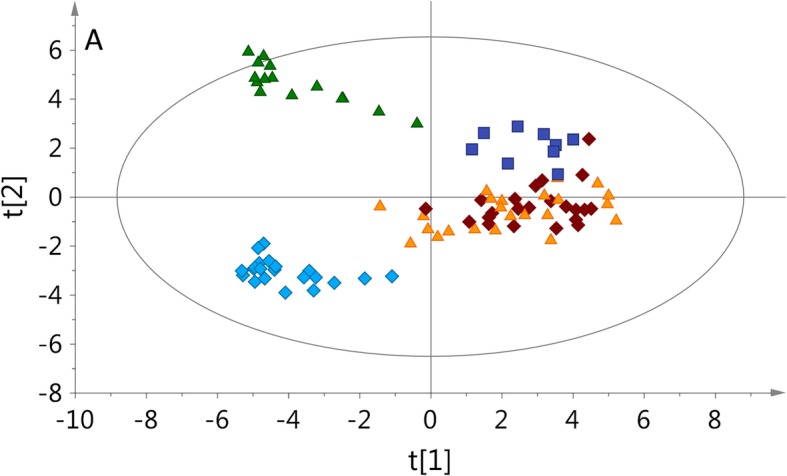
The Partial Least Squares - Discriminant Analysis model (R2 = 0.768; Q2 = 0.480) of the five investigated calf groups. 0 - born alive-control (blue rhombus), 1 - died after birth (orange triangle), 2 -died in utero, without signs of autolysis (brown rhombus), 3 - died in utero with mild to moderate autolysis (blue square), 4 - died in utero with gross autolysis (green triangle)

The PLS-DA model (Fig. 1) was calculated according to the obtained ^1^H NMR spectra. This model revealed a clear separation between the five sets of calf plasma samples (based on established PMI). The reference or control group, after normal calving (0), formed a well-separated compact group different from all other groups. Next we observed two sets that included calves that had died shortly after birth (1) or had died in utero, but without signs of autolysis (2). Calves that had died in utero with moderate autolysis (3) were shifted from the previous groups and formed the next group. The last group was represented by animals which had died in utero with gross autolysis, (4) and formed a separated and stretched cluster.

The same type of analysis was performed on the urine samples from the calves, but the results were unsatisfactory. Only a direct comparison between groups 0 and 4 yielded statistical significance in the calculated model (R^2^X = 0.53, Q^2^X = 0.77, *p* = 4.21 E-04). The direct mutual cross-comparison of all groups showed that the obtained PLS-DA models passed the validation procedure only for stage 4 vs. 0 over 1, 2, 3. However, the Q^2^ parameter, responsible for group separation, increased from 1 > 2 > 3, showing very distinct changes between these states.

**Fig. 2:**
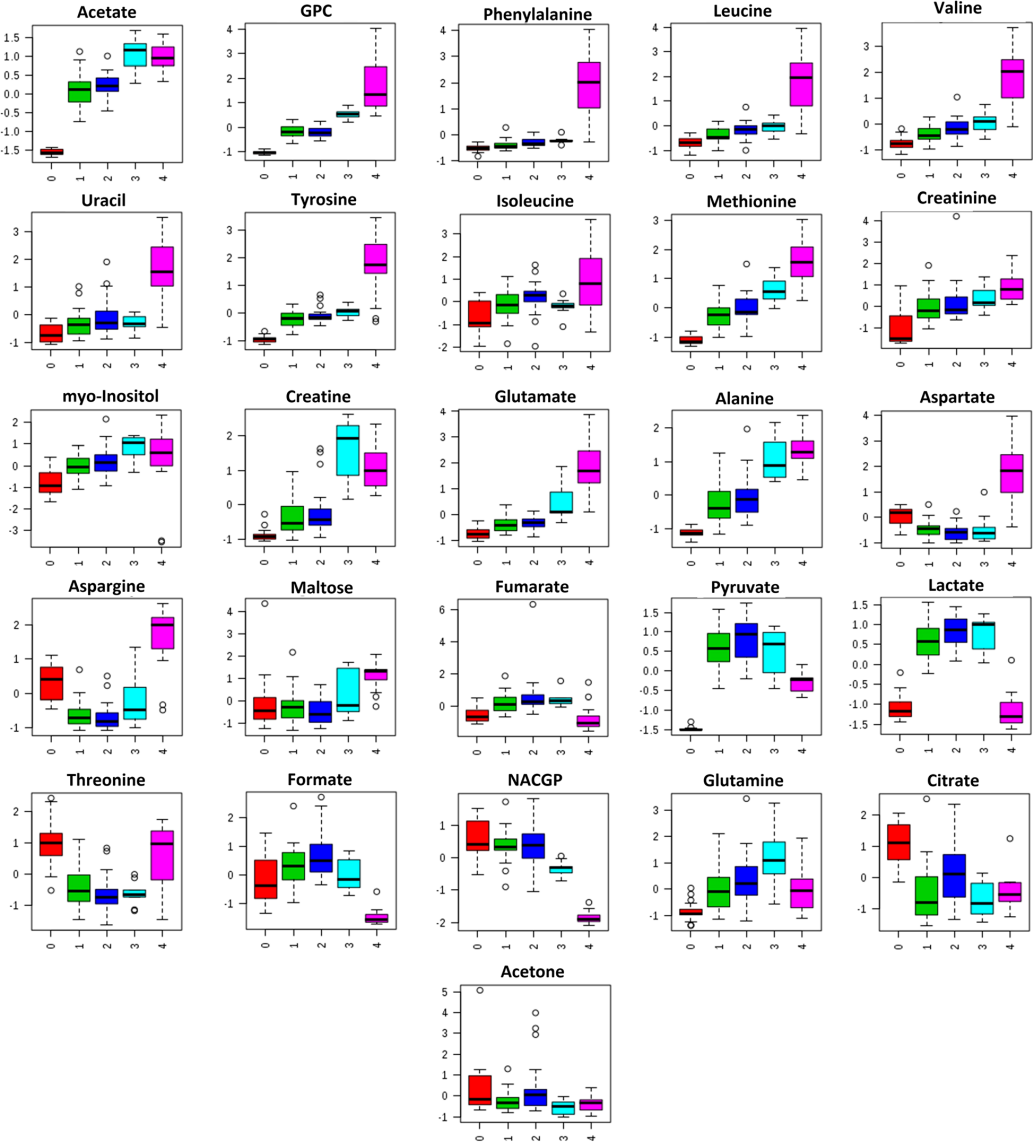
Calf plasma metabolites that have passed the one-way analysis of variance (ANOVA) test in the examined post-mortem interval groups. Median box-and-whisker plots representing the changes in relative integral values of the calf plasma metabolites. Groups (from left to right): 0 - born alive-control, 1 - died after birth, 2 - died in utero without signs of autolysis, 3 - died in utero with mild to moderate autolysis, 4 - died in utero with gross autolysis

To determine the relationship between the calf body condition and metabolism, reflected in the molecular compounds present in the plasma samples, ANOVA tests were performed for identified compounds and calf groups. This analysis, performed on metabolite levels, showed three types of changes (Fig. 2). The first type includes metabolites whose concentrations increased in plasma with PMI groups 1–4 (acetate, GPC (sn-glycero-3-phosphocholine), phenylalanine, leucine, valine, uracil, tyrosine, isoleucine, methionine, creatinine, myo-inositol, creatine, glutamate, alanine). The second type comprises the compounds whose relative concentrations decreased with increasing PMI (NAC (*N*-acetylated glycoproteins), citrate, acetone). The last type constitutes a heterogeneous group in which metabolites either decreased or increased with respect to the control group, and then subsequently increased or decreased respectively (initial decrease - aspartate, asparagine, maltose, and threonine; initial increase- fumarate, pyruvate, lactate, formate, and glutamine). The detailed statistical analysis of all changes found in the metabolites in plasma is given in Additional file 1: Table S1.

**Fig. 3:**
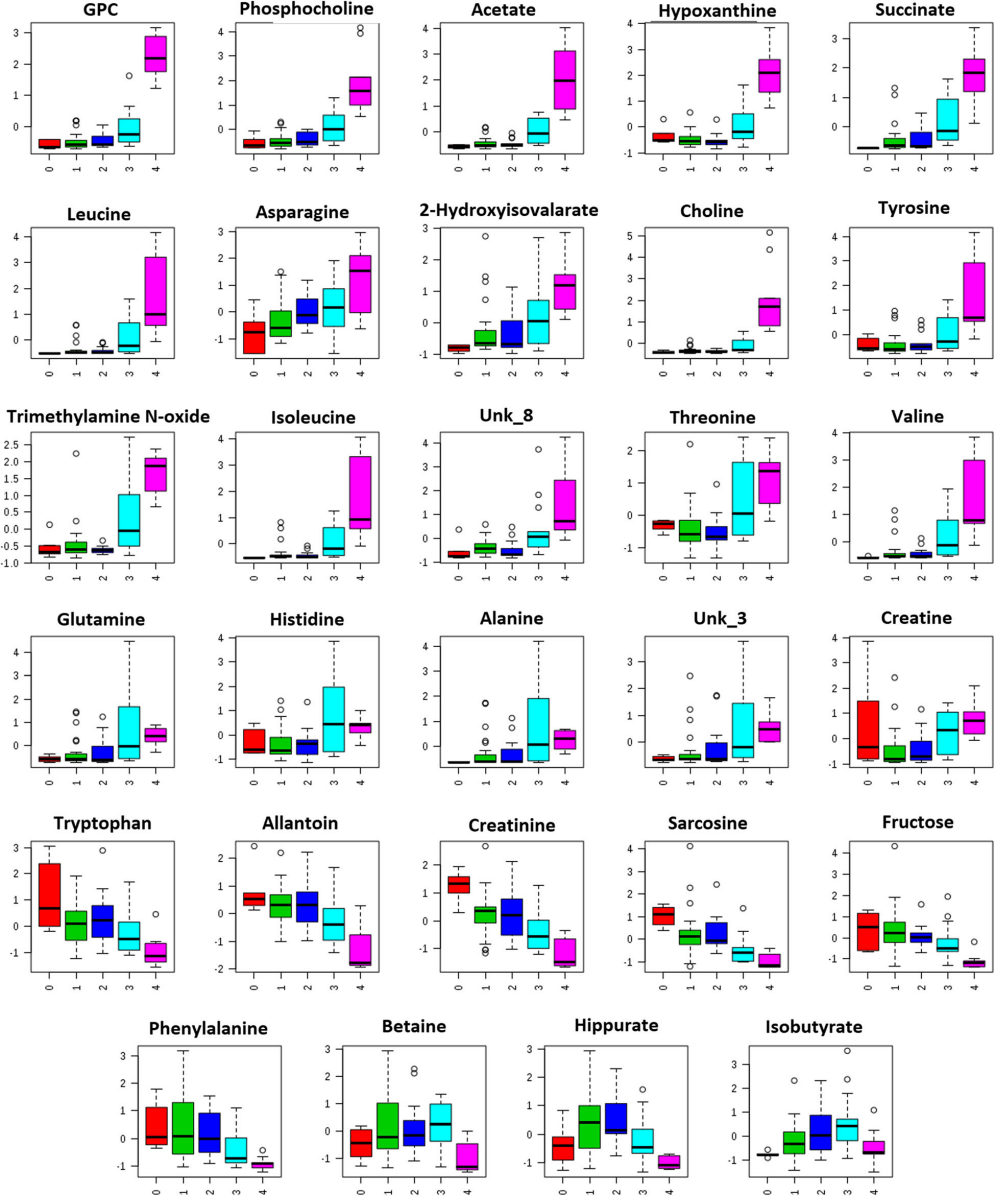
Calf urine metabolites that have passed an ANOVA test in the examined post-mortem interval groups. Median box-and-whisker plots representing the changes in relative integral values. Groups (from left to right): 0- born alive-control, 1- died after birth, 2- died in utero without signs of autolysis, 3- died in utero with mild to moderate autolysis, 4 - died in utero with gross autolysis

The analysis of calf urine metabolites also allowed the selection of 29 metabolites that differentiated between calf groups (Fig. 3). In this case, the metabolites were also divided into three groups: those whose concentrations tended to increase (GPC, phosphocholine, acetate, hypoxanthine, succinate, leucine, asparagine, 2-hydroxyisovalerate, choline, tyrosine, trimethylamine N-oxide, isoleucine, threonine, valine, glutamine, histidine, alanine, creatine, and two unknowns, Unk_3 and Unk_8), tended to decrease (tryptophan, allantoin, creatinine, sarcosine, fructose, and phenylalanine), and those that first increased and then decreased (betaine, hippurate, and isobutyrate). Detailed statistical analysis of all changes among the identified metabolites in urine is shown in Additional file 2: Table S2.

**Fig. 4:**
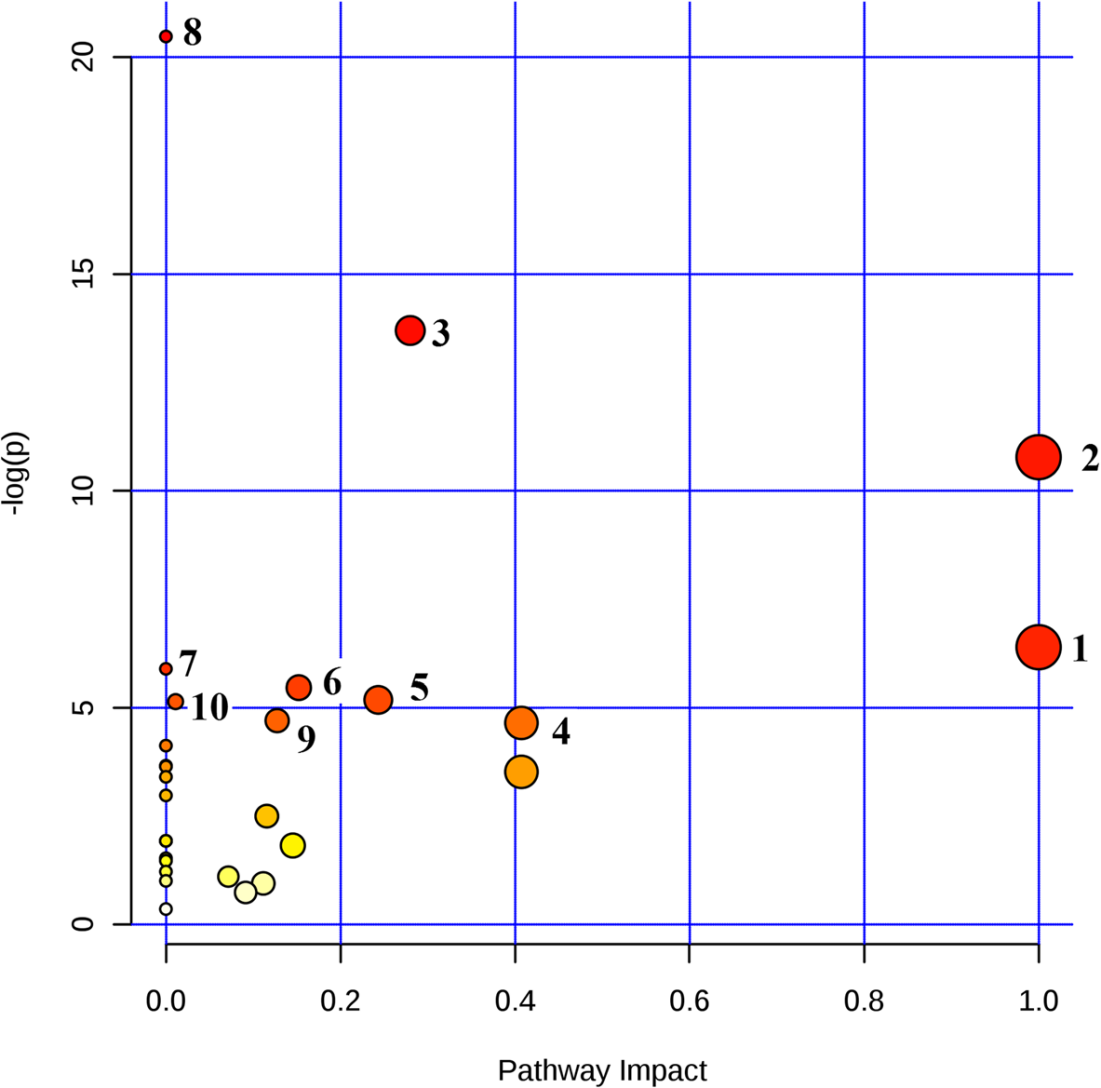
MetPA to identify the most perturbed biochemical pathways in calf plasma during post-mortem processes. 1 - Phenylalanine, tyrosine and tryptophan biosynthesis; 2 - Alanine, leucine and isoleucine biosynthesis; 3 - Alanine, aspartate and glutamate metabolism; 4 - Phenylalanine metabolism; 5 - Pyruvate metabolism; 6 - Citrate cycle (TCA cycle); 7 - D-Glutamine and D-glutamate metabolism; 8 - Aminoacyl-tRNA biosynthesis; 9 - Glycolysis or gluconeogenesis; 10 - Arginine and proline metabolism

**Fig. 5:**
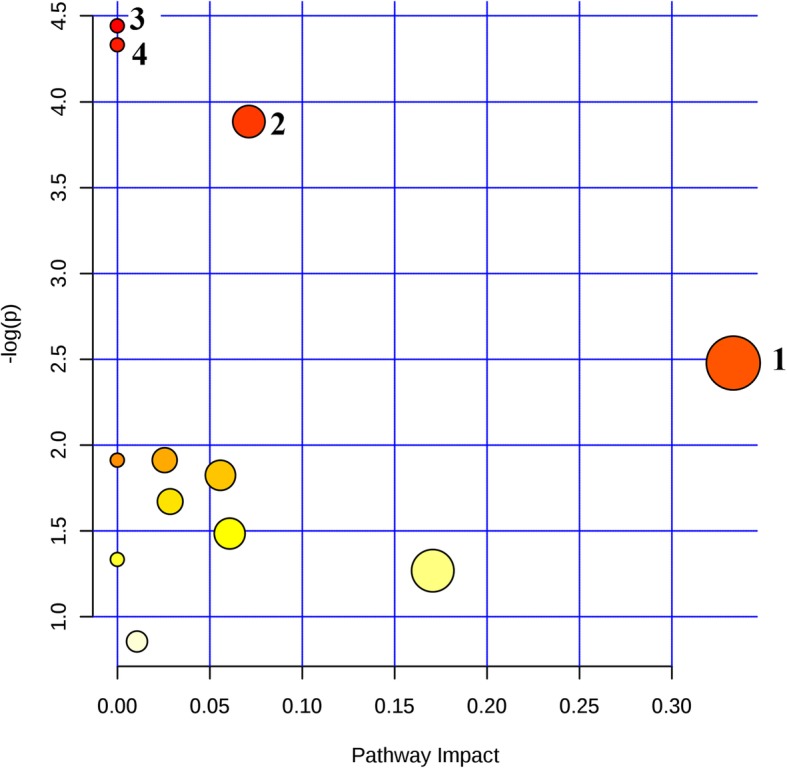
The MetPA within the most perturbed biochemical pathways in calf urine during post-mortem processes. 1 – Valine, leucine and isoleucine biosynthesis, 2 - Phenylalanine, tyrosine and tryptophan biosynthesis, 3 – Phenylalanine metabolism, 4 – Alanine, aspartate and glutamate metabolism

Changes in metabolites were best reflected by the MetPA (Figs. 4, 5), where the most perturbed pathways were assigned. In the comparison between the plasma and urine perturbed pathways, the common for these two biological compartments were the phenylalanine, tyrosine and tryptophan biosynthesis pathways.

## Discussion

The aim of this study was to evaluate if calves in different conditions at the time of calving would have different metabolomic profiles that may be used for evaluating the PMI. In calves which survived calving (0) or died relatively shortly after or before birth (1, 2), changes in plasma metabolites could be mainly due to hypoxia [7]. Post-mortem processes cause dramatic changes in cell and tissue metabolites and hence, in the biofluid content [8], and this would have had the greatest impact on changes observed in PMI groups 3 and 4. Estimating the impact of these processes on outcome is difficult, especially in the middle PMI groups (2 and 3). Taking all these variables into account, it is impossible to explain every single finding, and we can only speculate about the biochemical pathways involved.

During post-mortem processes, cell lysis, caused by membrane damage due to sodium ion accumulation, is provoked by dysfunction of the Na+/K+ pump [9, 10] resulting in uncontrolled enzyme action accompanying protein breakdown. There is post-mortem accumulation of certain metabolites not used for further biosynthesis cycles and no longer supplied for circulation; at the same time, some compounds are used in already ongoing processes without any further replenishment. All these processes could impact the pool of metabolites that change with time, in both the blood and the urine. Then, what is the source of metabolite content when internal fluid circulation is stopped and plasma metabolite contents are subjected to only blood cells and permeation from surrounding biofluids? The same question might be applied to urine taken directly from the bladder: are the changes local, or is some diffusion from the kidney still possible?

### Plasma metabolites

The analysis of the calf plasma samples showed an obvious difference in trend in metabolite levels between PMI groups 1–4, due to necropsy changes, and the control group. The stratification was based on the post-mortem changes observed during necropsy and in control calves (Fig. 1). However, based on PLS-DA, PMI groups 1 and 2 were similar to each other, but were distinctly different from the other groups (Fig. 1). The reason for this may be the very small difference in PMI time between these two groups, as the only difference between them was lung inflation. Group 2 probably experienced longer/deeper hypoxia/anoxia, which led to hypercapnia or acidosis followed by depression of the central nervous system that was severe enough to impair the reflexes that initiate respiration [11]. PMI 3 was different from stage 4 (Fig. 1) due to the ongoing changes that occurred in the environment of the uterus. It was probably the result of longer retention in the uterus after death which lead to most pronounced autolytic changes, as the calving-to-necropsy interval in all cases was relatively short.

The results show the main trend of continuous changes in the majority of parameters from the control group through PMI groups 1 to 4. Focusing on the metabolite content, the most typical blood changes with time after death are amino acids, lactic acid, hypoxanthine, uric acid, ammonia, NADH, and formic acid [8, 12]. Considering the metabolite content of the calves’ plasma in all groups, the most significant change was the increased level of amino acids which could originate from hypoxia [7], post-mortem protein degradation [13] due to cell lysis, and/or ongoing anabolic-catabolic processes, which can be strongly imbalanced post-mortem. However, all these processes may overlap. The decreasing trend in *N*-acetylated glycoproteins NACGP with PMI can be linked to advanced post-mortem processes accompanied by inhibited protein biosynthesis. An exception, to the trend in content level, were two mutually dependent amino acids, aspartate, and asparagine (Fig. 2), which were initially consumed (PMI 1, 2) and then exhibited high levels in PMI 4. This phenomenon may be explained by the conversion of aspartate to fumarate (which was increasing) by the TCA pathway until this route slowed, accompanied by the drop in fumarate at PMI 4. Another deviation from the general increased levels of amino acids was glutamine, which concentrations increased from PMI 1 to 3 and then significantly decreased at PMI 4 after being exhausted as an intermediate metabolite for ongoing biochemical processes. The increased plasma levels of branched-chain amino acids (leucine, isoleucine; Fig. 2) could be caused by all of these processes and are accompanied by limited gluconeogenesis processes, and protein degradation, with pyruvate initially increasing significantly (accumulation), and citrate diminishing (consumption), showing a general reversal to fumarate and, ultimately, a TCA slowdown. Interestingly, the course of changes between pyruvate and lactate may be related: the increase in lactic acid may be a response to ischemic-hypoxic conditions caused by skeletal muscle glycogen breakdown [14, 15]. Another mutual biochemical dependency was creatine (increased trend), which is associated with pyruvate via serine, glycine, and sarcosine pathways (KEGG glycine, serine, and threonine metabolism). Its high level can also be reflection of tissue lysis [16]. The increased level of creatinine in plasma with PMI groups could be due to cellular lysis from tissues and accumulation due to the inability of the kidneys to excrete it [12]. Similarly, myo-inositol, which is known to be involved in intracellular signalling mediation [17, 18] and membrane trafficking [19, 20], showed increased levels with PMI. Myo-inositol also functions in cell osmolyte regulation [21], where high levels can be a response to abnormal post-mortem conditions. Similarly, GPC can perform the same function [21] when released from perturbed cells. Another increased metabolite with PMI, uracil, could originate from the degradation of nucleoside or enzyme regulators, but its increased level cannot be explained easily. Acetate participates in many biochemical processes that occur in living organisms (glycolysis/gluconeogenesis, pyruvate metabolism, propanoate metabolism etc.); however, its positive correlation with pyruvate can be associated with downregulated TCA pathway activities. One more explanation for its increasing concentration described in literature is the post-mortem microbiome action [15, 16]. Due to a relatively short PMI in groups 1 and 2, this effect should be minimized in these groups, but we cannot exclude some level of bacterial activity in PMI groups 3 and 4. In healthy pre-partum cows, *Bacillus* sp., *Staphylococcus* sp., and *E. coli* were isolated from the vagina [22]. Dilatation of the cervix could enable microorganisms from the skin or faeces to enter the vagina and move to the uterus and decompose the fetus. Acetone is a product of lipid breakdown due to beta-oxidation and keto body degradation, and most probably decreased with PMI grade due to induction of ischemia-hypoxia accompanying conditions.

### Urine metabolites

Urine reflects the same three trends of changes as blood plasma; in the majority of metabolites, concentration was increasing with PMI, but in some we observed either decreasing or fluctuating trends. GPC and phosphocholine (raised values) are important intermediate metabolites in choline and glycerophospholipid metabolism. However, GPC, a known osmolyte [23, 24], that, especially in the kidneys, can protect against hypertonic stress-induced post-mortem processes, was found in this study to be significantly increased, not only in urine but also in plasma fluid. Additionally, GPC can be formed during phosphatidylcholine breakdown [25], and inhibiting GPC enzyme degradation can be a cause of its accumulation. The increasing concentration of 2-hydroxyisovalerate was related to lactic acidosis in calves as this metabolite was found in human urine, not only in keto but also in lactic acidosis [26]. The urine tryptophan and phenylalanine decrease could be caused by extensive use or limited supply after death or birth. At the same time, the increasing values of other amino acids (Asp, Tyr, Ile, Thr, Val, Gln, His) could indicate protein breakdown. However, all these processes are very difficult to correlate due to cessation/activation of enzyme action and hemolysis processes.

Hypoxanthine was found to be a marker of ATP breakdown [27] and is a key substance in purine metabolism. In the liver and intestine, it can be oxidized to uric acid [28] and, finally, to allantoin. In this study, hypoxanthine was found to be an important biomarker in urine, but not in the plasma and increased with PMI. In the study by Donaldson and Lamont [8] in rats, hypoxanthine concentration in blood increased with post-mortem time up to 48 h and then decreased, but in pigs the concentration in blood increased up to the end of the observation (96 h). Hypoxanthine oxidase is present in the blood of rats and cows but not in pigs, and its activity is higher in cows than in rats [29]. This might be the reason why hypoxanthine was not detected in calf plasma. Additionally, in humans, 58% of newborns with perinatal complications had elevated urinary excretion rates of hypoxanthine, and the increased values were suggested to be a retrospective marker for quantifying lack of oxygen [30]. This suggests that, in our study, calves at PMI 3 and 4 may have experienced the greatest oxygen depletion before they died and before filtration by the kidneys ceased. With increasing levels of hypoxanthine, we noticed a decreasing level of allantoin, a product of uric acid and a known antioxidant agent and scavenger of ROS (reactive oxygen species) and purine degradation metabolites [30–33]. Decreased concentration with increased PMI might show cessation of ROS action and/or decreased breakdown of hypoxanthine (despite its increase) to allantoin.

The level of succinate, a TCA metabolite, could be elevated due to reasons such as amino acid metabolism/catabolism due to muscle tissue breakdown in the putrefactive process, reductive carboxylation, or bacterial anabolic reactions [12]. Succinate was found to be a marker of kidney damage in diabetic patients [34]. Concentration of two coupled metabolites, creatinine, and sarcosine, decreased with increasing PMI, which may reveal the cessation of kidney activity and processes occurring in tissues surroundings the bladder. Interestingly, in calves, urine fructose was found to decrease with PMI. This phenomenon can be related to the cell energy demand and/or lack of further supply. Among the metabolites that showed variable changes, we identified hippurate which is related to microflora activity, liver function, and renal malfunction [35] and all these factors might have significant roles in this study. However, the fluctuations in hippurate concentration may be in response to the different post-mortem processes occurring in stages 1 and 2 and the ceasing of the kidney functions in PMI groups 3 and 4. A similar variation in dependence of metabolite level on PMI was exhibited by betaine and isobutyrate also, which increased from 0 to 3 PMI and decreased in group 4. Betaine is known as a kidney protectant (osmolyte) whose level increases with hypertonicity [36], which may be the cause of urine permeating the kidney. Betaine is also tightly connected to choline metabolism, which comes from glycerophospholipid metabolism and, in this case, is positively correlated up to stage 3; for stage 4, the reverse trend is observed. The last metabolite whose concentration fluctuated within the PMIs was isobutyrate. Production of this compound from amino acids may be associated with the activity of microflora (such as yeasts, *Enterococci* or *Clostridia*) [37].

## Conclusions

In this work, post-mortem changes were associated with molecular variations in calf blood plasma and urine. There was a visible trend of changes in metabolites from the control group (0) to PMI group 4. The analysis of metabolite changes with increasing PMI groups shows, in general, the active and slowly terminating biochemical pathways. The results of the study show that evaluation of calf plasma or urine may be used as a tool for determination of PMI. Moreover, the metabolites, which unambiguously increased or decreased, can be used as potential biomarkers of PMI.

## Materials and methods

### Animals and sampling procedures

The material was collected from November 2013 to June 2015 in 20 Polish Holstein-Friesian herds (1–1037 cows/herd; median: 185 cows/herd). The cubicle housing system was present in 13 herds and the tied-up system in 7 herds. In 15 herds, cows were fed with total mixed rations and in 5 herds, the ingredients were fed separately. The herds were from the southwestern part of Poland. For convenience, herds located within a one-way driving distance of 2.5 h from the Wroclaw University of Environmental and Life Scienceswere recruited.

Out of 121 SB calves, only 75 calves were enrolled in the study due to insufficient sample availability. The SB calves were born dead or died within 6 h of birth. None of the calves received colostrum. The SB calves were either Holstein-Friesian (*n* = 70) or Holstein-Friesian crossbreeds (*n* = 5) comprising Simental, Jersey, Limousin, and Brown Swiss Sires. All necropsies of SB calves were performed 8 ± 3 h after calving. The post-mortem examination was performed in the necropsy room at Wroclaw University of Environmental and Life Sciences. All carcasses were subjected to systematic gross external and internal examinations according to the same project-specific protocol. During necropsy of the calves, blood from the jugular vein or aorta was aseptically collected in syringes and immediately divided into 5 mL heparinized tubes (MEUS Srl®, Piove di Sacco, Italy, 18,648); 3–5 mL of urine sample (urocentesis), if available, was collected with a sterile syringe.

Twenty one calves, born after a normal pregnancy length (≥260 days), singleton and with assisted calving (6 calves without and 15 with a calving jack) were assigned to the control group. All the control calves were Holstein-Friesian. Blood from the control calves was collected from the jugular vein. Six male calves from control group were premedicated with 1 mL (20 mg) xylazine (Sedazin®, Biowet Pulawy) and 1 mL (100 mg) ketamine IV (Bioketan®, Vetoquinol) and then euthanized with a mixture of pentobarbital and pentobarbital sodium, 160 mg/mL IV (Morbital®, Biovet Pulawy) at a dose of 48–96 mg/kg body weight. These calves were examined according the same protocol as SB calves. Necropsies of control calves were performed within 6 ± 3 h after calving and within 2 ± 0.67 h after death.

All plasma and urine samples were centrifuged (14 min at 1860 g), aliquoted and frozen at − 80 °C until analysis. Samples were analyzed within 6 months of collection.

Based on gross post-mortem changes, the SB calves were divided into four PMI groups:

1. Calves that died after birth with partial (at least one third) or full lung inflation, were assigned to PMI group 1.
2. Calves that died in utero with lungs not inflated or inflation area not exceeding one third of lung area and without signs of autolysis in internal organs, were assigned to group 2.
3. Calves that died in utero with mild to moderate autolysis (presented friability and softening of internal organs - kidney, spleen, brain), and hemoglobin imbibition in the pleura and aortic arch, were assigned to group 3.
4. Calves that died in utero with gross autolysis, presented liquefaction of internal organs (kidney, spleen, brain) and uniform, marked homogenous hemoglobin imbibition in the pleura, aortic arch, and all body tissues, were assigned to group 4.
5. Calves from the control group were tagged as group 0.

The number of samples examined in each group is presented in Table 1.

**Table 1:** The number of samples of blood plasma and urine accordingly to PMI

	PMI				
	0	1	2	3	4
Calves Blood Plasma	21	24	23	11	17
Calves Urine	6	13	23	9	16

There are no duplicates of samples from the same individuals. Groups: 0- born alive-control, 1- died after birth, 2- died in utero, without signs of autolysis, 3- died in utero with mild to moderate autolysis, 4- died in utero with gross autolysis

### Sample preparation and ^1^H NMR measurements

The urine and calf blood plasma were prepared and measured according to earlier described procedures Wojtowicz et al. [38].

### Preprocessing of variables prior to analysis

The metabolite resonances (listed in Additional file 1: Table S1 and Additional file 2: Table S2) were identified according to the assignments published in literature and in on-line databases (Biological Magnetic Resonance Data Bank and Human Metabolome Data Base). For quantification purposes, integrals of the non-overlapping signal fragments were used. All the variables (originating from different fluids) were scaled to unit standard deviation.

### Multivariate data analysis

Multivariate data analysis was performed using SIMCA software (v 14.0, Umetrics). The order of the samples in the dataset was randomized. The discriminant version of the PLS-DA with a default k-fold cross-validation procedure was used to determine the differences between the groups.

### Statistical data analysis

For each metabolite in the measured samples, the percentage difference (PD) and relative standard deviation were calculated using STATISTICA 12. PD was calculated based on the mean values of the relative signal integrals in each group. The calculations were performed from left to right. For group comparisons of the relationship between calf body condition and metabolism, i.e. molecular compounds present in plasma and urine samples, ANOVA with box-and-whisker plots was performed. All calculations were carried out by using the multitools platform for metabolomics analysis on the website https://www.metaboanalyst.ca/. ANOVA was used to determine whether there were any statistically significant differences between the means of two or more independent (unrelated) groups.

For the metabolites chosen, normality was tested with the Shapiro-Wilk test, and then on the basis of the obtained results, the statistical significance, based on the *Mann*–*Whitney*–Wilcoxon (*p* < 0.05) or Student’s t-test (*p* < 0.05), was calculated.

### Pathway analysis – MetPa

For metabolite data analysis, the MetaboAnalyst 3.0 platform was used with selected features. Pathway analysis was performed on a relative integral matrix with only the identified metabolites [39].

## Additional files

Additional file 1: **Table S1**. Changes among metabolites of blood plasma samples. (DOCX 34 kb)

Additional file 2: **Table S2**. Changes among metabolites of urine samples. (DOCX 36 kb)

## Data Availability

The datasets used and analyzed during the current study are available from the corresponding author on reasonable request.

## Abbreviations

^1^H NMR: proton nuclear magnetic resonance
ANOVA: one-way analysis of variance
GPC: sn-glycero-3-phosphocholine
NAC: *N*-acetylated glycoproteins
NACGP: *N*-acetylated glycoproteins
NMR: nuclear magnetic resonance
PD: percentage difference
PLS-DA: Partial Least Squares - Discriminant Analysis
PMI: post-mortem interval
SB: stillborn
TCA: tricarboxylic acid cycle
TSP: trimethylsilyl-2,2,3,3-tetradeuteropropionic acid
