# Supplementary material for: Metabolomic studies as a tool for determining the post-mortem interval (PMI) in stillborn calves
Source: BMC Vet Res. 2019 Jun 7;15:189. doi: 10.1186/s12917-019-1935-4 (PMC6555048; doi:10.1186/s12917-019-1935-4)
Supplement: Supplementary file 1 — Table S1. Changes among metabolites of blood plasma samples. (DOCX 34 kb) [file 12917_2019_1935_MOESM1_ESM.docx]

Additional file 1: Table S1. Changes among metabolites of blood plasma samples.

| **Metabolite** | **Percentage difference** | | | | | | | | | | **Relative standard deviation [%]** | | | | |
| --- | --- | --- | --- | --- | --- | --- | --- | --- | --- | --- | --- | --- | --- | --- | --- |
|  | **0 vs 1** | **0 vs 2** | **0 vs 3** | **0 vs 4** | **1 vs 2** | **1 vs 3** | **1 vs 4** | **2 vs 3** | **2 vs 4** | **3 vs 4** | **0** | **1** | **2** | **3** | **4** |
| Isolecine | -13,15* | -16,46* | -9,34 | -29,36* | -3,33 | 3,82 | -16,37* | 7,14 | -13,06 | -20,16* | 15,51 | 16,24 | 13,20 | 8,47 | 28,25 |
| Leucine | -18,67* | -26,40* | -33,06* | -83,93* | -7,83 | -14,62* | -67,92* | -6,81 | -60,90* | -54,66* | 14,93 | 17,37 | 16,57 | 13,31 | 36,21 |
| Valine | -26,03* | -35,29* | -46,57* | -94,82* | -9,47 | -21,17* | -73,31* | -11,76 | -64,96* | -54,23* | 18,66 | 19,23 | 21,21 | 19,16 | 34,27 |
| Alanine | -77,10* | -89,39* | -122,28* | -123,28* | -14,84 | -59,11* | -60,58* | -45,26* | -46,79* | -1,62 | 18,62 | 38,27 | 36,93 | 23,33 | 26,47 |
| Acetate | -104,62* | -106,71* | -126,39* | -122,02* | -2,89 | -32,51* | -25,55* | -29,69* | -22,70* | 7,11 | 10,72 | 18,22 | 12,50 | 13,07 | 17,59 |
| NAC | 4,37 | 6,85 | 29,24* | 95,68* | 2,48 | 24,95* | 92,28* | 22,51* | 90,32* | 71,44* | 16,60 | 17,80 | 16,87 | 8,80 | 46,31 |
| Methionine | -51,41* | -59,97* | -81,37* | -100,48* | -9,28 | -33,46* | -56,35* | -24,37* | -47,69* | -24,02* | 11,85 | 18,96 | 22,30 | 16,00 | 26,80 |
| Acetone | 28,55* | -0,61 | -45,22 | 32,00* | -29,14* | -71,46* | 3,53 | -44,64 | 32,59* | 74,52 | 50,81 | 24,74 | 51,23 | 121,13 | 23,29 |
| Glutamate | -21,24* | -20,50* | -52,36* | -83,53* | 0,75 | -32,01* | -65,18* | -32,74* | -65,85* | -34,99* | 14,06 | 15,79 | 10,58 | 25,13 | 28,35 |
| Pyruvate | -175,16* | -177,05* | -173,78* | -161,05* | -8,40 | 5,79 | 47,89* | 14,17 | 55,73* | 42,39* | 30,52 | 24,76 | 24,78 | 28,21 | 22,73 |
| Glutamine | -27,32* | -39,13* | -58,31* | -24,36* | -12,13 | -32,28* | 3,01 | -20,34* | 15,13 | 35,20* | 13,67 | 23,16 | 25,88 | 24,49 | 25,15 |
| Citrate | 44,64* | 25,98* | 50,63* | 42,19* | -19,21* | 6,35 | -2,57 | 25,48* | 16,67 | -8,91 | 14,93 | 35,96 | 27,34 | 22,37 | 20,81 |
| Aspartate | 70,15* | 80,30* | 61,51* | -84,91* | 11,81 | -9,68 | -134,96* | -21,43 | -141,15* | -129,51* | 34,03 | 70,90 | 72,09 | 109,50 | 46,15 |
| Asparagine | 102,46* | 107,23* | 39,25 | -60,72* | 6,58 | -70,27* | -141,21* | -75,98* | -144,44* | -94,34* | 36,95 | 95,30 | 95,54 | 90,10 | 37,94 |
| Creatine | -66,49* | -78,64* | -139,68* | -126,74* | -13,97 | -95,33* | -76,32* | -84,16* | -64,06* | 23,23 | 34,01 | 42,95 | 56,59 | 31,48 | 33,57 |
| Creatinine | -60,52* | -69,59* | -74,20* | -87,64* | -10,13 | -15,40 | -31,26* | -5,29 | -21,30 | -16,05 | 62,06 | 32,31 | 42,02 | 21,65 | 24,94 |
| Choline | 6,48 | -13,39 | -6,09 | -31,59 | -19,83 | -12,56 | -37,87* | 7,31 | -18,39 | -25,62* | 82,74 | 34,47 | 39,36 | 24,74 | 25,86 |
| GPC | -98,19* | -93,92* | -125,88* | -147,38* | 5,55 | -40,08* | -77,07* | -45,37* | -81,75* | -40,09* | 14,00 | 20,15 | 16,37 | 11,86 | 38,46 |
| myo-Inositol | -25,66* | -26,73* | -41,25* | -29,41* | -1,09 | -16,01* | -3,83 | -14,93 | -2,74 | 12,20 | 18,58 | 13,96 | 18,20 | 12,87 | 41,44 |
| Lactate | -103,72* | -109,35* | -107,07* | 1,89 | -7,86 | -4,64 | 105,09* | 3,23 | 110,66* | 108,41* | 39,43 | 20,21 | 12,85 | 16,93 | 65,80 |
| Threonine | 59,06* | 71,01* | 70,83* | 12,18 | 13,35 | 13,15 | -47,74* | -0,20 | -60,13* | -59,95* | 24,80 | 47,41 | 40,04 | 27,57 | 39,00 |
| Maltose | 5,10 | 28,03 | -28,90 | -67,57* | 23,01 | -33,87 | -72,05* | -55,79* | -91,28* | -40,66* | 104,39 | 73,81 | 63,74 | 67,76 | 32,19 |
| Glucose | -44,33* | -12,58 | -39,04 | 9,95 | 32,20 | 5,53 | 53,69* | -26,79 | 22,46 | 48,52 | 69,05 | 64,40 | 68,30 | 101,85 | 73,06 |
| Uracil | -53,14* | -73,92* | -56,34* | -137,91* | -23,04 | -3,46 | -103,79* | 19,62 | -85,88* | -101,23* | 61,67 | 50,86 | 63,46 | 30,13 | 39,04 |
| Fumarate | -45,46* | -62,34* | -53,96* | 8,52 | -18,16 | -9,05 | 53,47* | 9,15 | 69,93* | 61,77* | 30,57 | 29,11 | 50,33 | 19,49 | 62,35 |
| Tyrosine | -92,84* | -99,59* | -106,96* | -149,20* | -8,79 | -18,78* | -86,22 | -10,04 | -78,92* | -70,28* | 28,50 | 21,10 | 20,11 | 14,68 | 43,36 |
| Phenylalanine | -18,09* | -25,39* | -33,92* | -118,57* | -7,39 | -16,07* | -106,17* | -8,71 | -100,76* | -94,11* | 16,69 | 22,23 | 17,01 | 12,04 | 47,28 |
| Formate | -22,65 | -26,34* | -5,12 | 92,79* | -3,75 | 17,58 | 109,68* | 21,30 | 112,28* | 96,76* | 43,75 | 35,40 | 25,47 | 26,10 | 80,97 |

Groups: 0 - born alive-control, 1 - died after birth, 2 - died in utero, without signs of autolysis, 3 - died in utero with mild to moderate autolysis, 4 - died in utero with gross autolysis. Significant differences marked with * at *p*<0.05.
