# Supplementary material for: Metabolomic studies as a tool for determining the post-mortem interval (PMI) in stillborn calves
Source: BMC Vet Res. 2019 Jun 7;15:189. doi: 10.1186/s12917-019-1935-4 (PMC6555048; doi:10.1186/s12917-019-1935-4)
Supplement: Supplementary file 2 — Table S2. Changes among metabolites of urine samples. (DOCX 36 kb) [file 12917_2019_1935_MOESM2_ESM.docx]

Additional file 2: Table S2. Changes among metabolites of urine samples.

| **Metabolite** | **Percentage difference** | | | | | | | | | | **Relative standard deviation [%]** | | | | |
| --- | --- | --- | --- | --- | --- | --- | --- | --- | --- | --- | --- | --- | --- | --- | --- |
|  | **0 vs 1** | **0 vs 2** | **0 vs 3** | **0 vs 4** | **1 vs 2** | **1 vs 3** | **1 vs 4** | **2 vs 3** | **2 vs 4** | **3 vs 4** | **0** | **1** | **2** | **3** | **4** |
| 2-Hydroxyisovalerate | -75.448 | -65.61 | -103.768* | -138.823* | 11.228 | -35.212 | -85.856* | -45.985 | -94.799* | -54.785* | 47.634 | 98.542 | 82.251 | 76.613 | 39.765 |
| 3-Hydroxybutyrate | -59.124 | -62.269 | -105.171* | *-93.173 | -3.463 | -54.523* | -39.487* | -51.302* | -36.148 | 15.891 | 41.483 | 109.993 | 108.696 | 82.641 | 65.147 |
| 3-Hydroxyisobutyrate | -99.716* | -117.291 | -137.676* | -110.037* | -24.837 | -57.796 | -14.223 | -34.186 | 10.709 | 44.488 | 87.107 | 120.399 | 110.558 | 78.377 | 57.002 |
| Acetate | -19.41 | -11.525 | -83.799* | -153.67* | 7.929 | -67.118* | -145.078* | -74.062* | -148.73* | -103.045* | 38.266 | 50.502 | 41.047 | 51.217 | 42.235 |
| Acetone | -29.41 | 2.081 | -88.589 | 26.397 | 31.442 | -63.304 | 54.743 | -90.254 | 24.349* | 108.635 | 39.354 | 129.315 | 35.275 | 196.115 | 22.257 |
| Alanine | -116.522 | -112.05 | -170.395* | -157.24* | 6.638 | -106.971* | -75.133* | -111.627* | -80.764* | 39.843 | 122.427 | 167.314 | 148.173 | 106.409 | 40.421 |
| Allantoin | 11.616 | 8.413 | 38.442* | 108.889* | -3.211 | 27.128* | 100.449* | 30.273 | 102.831* | 78.682* | 29.292 | 33.395 | 33.815 | 47.825 | 102.648 |
| Ascorbate | -12.324 | 2.321 | -25.326 | -8.637 | 14.634 | -13.104 | 3.697 | -27.606 | -10.953 | 16.78 | 29.978 | 98.508 | 99.009 | 58.244 | 23.864 |
| Asparagine | -42.403 | -65.683* | -73.583* | -110.103* | -25.022 | -33.818 | -76.646* | -8.986 | -54.224* | -45.796 | 89.184 | 63.609 | 38.57 | 59.482 | 47.033 |
| Betaine | -33.106 | -32.129 | -34.144* | 31.158 | 1.003 | -1.068 | 62.649* | -2.071 | 61.742* | 63.61* | 37.672 | 45.877 | 43.007 | 31.941 | 47.667 |
| Citrate | -35.427 | -73.535 | -71.097* | 34.773 | -40.762 | -38.067 | 68.103 | 2.804 | 101.8 | 99.708* | 144.527 | 123.889 | 122.13 | 85.51 | 87.976 |
| Choline | -14.577 | 8.85 | -82.278* | -175.264* | 23.352 | -69.794* | -171.65* | -89.499* | -177.241* | -145.406* | 105.681 | 76.566 | 42.552 | 78.258 | 66.194 |
| Creatine | 65.081 | 56.442 | -7.157 | -32.664 | -9.513 | -71.407* | -92.813* | -62.963* | -85.18* | -25.658 | 118.118 | 122.084 | 94.507 | 56.718 | 36.023 |
| Creatinine | 32.511* | 27.336 | 65.819* | 127.079* | -5.293 | 35.19* | 105.461* | 40.296* | 109.23* | 77.457* | 20.977 | 40.885 | 44.347 | 50.148 | 83.403 |
| Formate | -79.559 | -36.059 | -3.401 | -16.384 | 46.861 | 76.676* | 65.303* | 32.758 | 19.97 | -13.001 | 63.178 | 100.385 | 71.842 | 71.322 | 172.759 |
| Fructose | -16.394 | 2.715 | 22.927 | 106.918* | 19.088 | 38.955* | 118.136* | 20.244 | 104.965* | 89.474* | 43.375 | 54.879 | 32.193 | 59.341 | 68.434 |
| Fumarate | -47.769* | -78.135* | -104.883* | -52.332* | -33.492 | -65.292 | -4.867 | -33.64 | 28.741 | 60.909 | 296.668 | 191.869 | 153.644 | 94.046 | 134.443 |
| Glucose | -39.836 | 26.977 | 0.56 | -89.038 | 65.065 | 40.374 | -53.989* | -26.427 | -109.443* | -89.487 | 26.943 | 145.383 | 53.094 | 48.832 | 94.376 |
| Glutamine | -34.09 | -32.754 | -106.225* | -92.926* | 1.374 | -79.316* | -63.897* | -80.471* | -65.128* | 17.656 | 65.302 | 92.436 | 88.977 | 91.423 | 28.027 |
| Hippurate | -31.704 | -37.162* | -0.257 | 76.012* | -5.624 | 31.453 | 101.595* | 36.914* | 105.709* | 76.232* | 42.677 | 55.672 | 37.933 | 51.3 | 34.135 |
| Histidine | -11.251 | -9.926 | -75.927 | -59.15* | 1.329 | -66.088 | -48.71* | -67.269 | -49.957* | 18.899 | 50.914 | 66.414 | 63.618 | 76.294 | 27.85 |
| Hypoxanthine | 32.851 | 51.042 | -46.419 | -135.404* | 18.987 | -76.359* | -151.417* | -92.01* | -158.977* | -105.574* | 85.865 | 76.134 | 89.022 | 78.887 | 32.273 |
| Isobutyrate | -48.421* | -72.605 | -85.591* | -31.497 | -26.514 | -41.466* | 17.595 | -15.375 | 43.6 | 58.003* | 52.19 | 59.176 | 60.064 | 55.611 | 63.232 |
| Isoleucine | -103.277* | -56.18 | -159.931* | -188.003* | 55.087 | -96.503* | -164.647* | -133.807* | -179.119* | -113.05* | 109.432 | 161.064 | 116.738 | 103.09 | 69.576 |
| Lactate | -101.499* | -99.486* | -151.045* | -138.429* | 2.692 | -80.337* | -56.926* | -82.582* | -59.39* | 26.433 | 247.238 | 172.804 | 154.194 | 102.921 | 63.943 |
| Leucine | -72.487* | -40.979 | -142.605* | -181.072* | 34.035 | -94.553* | -161.617* | -119.013* | -171.999* | -108.523* | 81.354 | 133.146 | 89.563 | 101.055 | 68.486 |
| N.N-Dimethylglycine | -44.422 | -51.72 | -69.469* | 24.474 | -7.743 | -27.141 | 67.073* | -19.5 | 73.857* | 90.112* | 61.835 | 77.277 | 65.05 | 60.866 | 81.105 |
| O-Phosphocholine | 11.102 | -3.625 | -68.614* | -143.687* | -14.712 | -78.226* | -148.852* | -65.395* | -141.909* | -99.628* | 97.272 | 74.775 | 58.177 | 66.957 | 45.203 |
| Oxypurinol | 26.256 | 45.458 | -62.68 | -112.295* | 19.793 | -85.422* | -129.04* | -100.947* | -139.899* | -60.21* | 112.006 | 74.858 | 103.674 | 96.011 | 66.026 |
| Pantothenate | -2.193 | 3.249 | -17.003 | 61.191* | 5.44 | -14.824 | 63.171* | -20.223 | 58.232* | 76.211* | 39.327 | 55.104 | 26.816 | 63.816 | 16.326 |
| Phenylalanine | 6.857 | 21.456 | 56.381* | 103.163* | 14.653 | 50.008* | 98.04* | 36.014* | 86.493* | 54.741* | 43.017 | 59.869 | 44.996 | 58.238 | 34.503 |
| Proline | -61.299 | -27.31 | -71.842 | -58.555* | 35.474 | -11.847 | 3.015* | -46.83 | -32.546 | 14.849 | 85.597 | 184.511 | 42.557 | 70.725 | 31.933 |
| Pyruvate | -89.069 | -105.686 | -138.672 | -61.163 | -21.731 | -71.762 | 32.306 | -52.06 | 53.106 | 98.367 | 89.386 | 161.206 | 138.121 | 116.432 | 122.36 |
| Sarcosine | 19.861 | 17.987 | 71.965* | 118.419* | -1.89 | 54.035* | 104.715* | 55.783* | 106.08* | 59.03* | 32.152 | 62.622 | 43.185 | 59.803 | 57.89 |
| sn-Glycero-3-phosphocholine | -17.977 | -22.89 | -92.35* | -166.191* | -4.964 | -77.593* | -160.178* | -73.335* | -158.362* | -119.812* | 91.978 | 90.576 | 70.719 | 80.653 | 21.269 |
| Succinate | 10.731* | 15.578 | -73.791* | -140.477* | 4.868 | -82.881* | -145.716* | -86.872* | -147.96* | -90.013* | 233.625 | 133.175 | 105.663 | 80.45 | 38.754 |
| Taurine | 6.862 | 36.25 | -0.917 | 7.086 | 29.572 | -7.777 | 0.225 | -37.136 | -29.352 | 8.002 | 75.054 | 131.307 | 74.461 | 60.333 | 49.556 |
| Threonine | 18.725 | 32.969 | -45.919 | -69.13* | 14.467 | -63.284* | -85.102* | -76.012* | -96.596* | -25.212 | 45.898 | 77.379 | 70.693 | 66.746 | 36.085 |
| Trimethylamine N-oxide | 6.222 | 51.73 | -78.304* | -128.527* | 45.877 | -83.509* | -132.108* | -118.077* | -154.566* | -67.108* | 107.094 | 120.148 | 32.267 | 82.603 | 23.246 |
| Tryptophan | 22.109 | 11.623 | 36.198* | 68.761* | -10.554 | 14.376 | 48.495* | 24.836 | 58.303* | 34.724* | 37.739 | 31.309 | 35.722 | 35.393 | 40.081 |
| Tyrosine | -10.28 | -17.064 | -68.611 | -132.312* | -6.814 | -59.378* | -126.328* | -53.101 | -122.142* | -82.402* | 47.682 | 90.453 | 72.339 | 74.831 | 62.882 |
| Valine | -93.567 | -57.821 | -155.7* | -182.383* | 41.337 | -97.726* | -154.901* | -126.307* | -169.159* | -91.987* | 124.023 | 162.951 | 121.388 | 102.687 | 66.167 |
| Xanthine | 56.964* | 43.819 | 43.828* | 97.288* | -14.02 | -14.01 | 46.809 | 0.009 | 59.847 | 59.839 | 45.526 | 57.527 | 54.996 | 96.374 | 89.325 |
| Tau-Methylhistidine | -56.288 | -81.219 | -81.202 | -77.411* | -28.149 | -28.128 | -23.705 | 0.021 | 4.519 | 4.498 | 267.832 | 152.296 | 110.273 | 87.85 | 60.085 |
| Unk_1 | -34.935 | -47.11 | -85.691* | -71.362* | -12.698 | -54.862* | -38.848* | -42.912 | -26.477 | 16.916 | 26.844 | 70.694 | 73.024 | 73.784 | 41.751 |
| Unk_2 | -57.229 | -69.413 | -112.687* | -89.839* | -13.528 | -66.118* | -37.42* | -53.793 | -24.199 | 30.59 | 28.89 | 92.509 | 92.661 | 82.807 | 57.412 |
| Unk_3 | -32.896 | -44.564 | -84.227* | -86.807* | -12.113 | -55.152* | -58.056* | -43.77 | -46.765* | -3.157 | 19.312 | 76.407 | 76.995 | 76.379 | 33.633 |
| Unk_4 | -58.177 | -56.121 | -107.292* | -88.511* | 2.239 | -58.196* | -34.815* | -60.239* | -36.982* | 24.628 | 51.44 | 104.532 | 88.541 | 78.949 | 45.804 |
| Unk_5 | -42.844 | -44.932 | -100.533* | -94.103* | -2.194 | -64.651* | -57.004* | -62.68* | -54.983* | 8.423 | 94.889 | 118.161 | 101.363 | 86.817 | 43.707 |
| Unk_6 | -4.611 | -5.237 | -35.276 | 3.089 | -0.626 | -30.79 | 7.697 | -30.178 | 8.322 | 38.26 | 30.169 | 36.497 | 26.459 | 43.734 | 45.552 |
| Unk_7 | 28.775 | 21.869 | 43.306* | 83.041* | -7.017 | 14.999 | 57.714 | 21.957 | 64.082 | 43.66 | 64.304 | 123.675 | 87.686 | 172.945 | 186.665 |

Groups: 0 - born alive-control, 1- died after birth, 2 - died in utero, without signs of autolysis, 3 - died in utero with mild to moderate autolysis, 4 - died in utero with gross autolysis. Significant differences marked with * at *p*<0.05.
